# Comprehensive 3D mapping reveals distinct spatial gradients of genetically-identified SST, PV, and TH interneurons across the mouse caudoputamen

**DOI:** 10.3389/fncel.2026.1795921

**Published:** 2026-04-16

**Authors:** Jonibek M. Muhsinov, Evan A. Iliakis, Wenxin Tu, Alexandra N. Ramirez, Michael A. Muniak, Andrzej Z. Wasilczuk, M. Felicia Davatolhagh, Alex Proekt, Tianyi Mao, Marc Vincent Fuccillo

**Affiliations:** 1 Department of Neuroscience, Perelman School of Medicine, University of Pennsylvania, Philadelphia, PA, United States; 2 Nash Family Department of Neuroscience, Icahn School of Medicine at Mount Sinai, New York, NY, United States; 3 Vollum Institute, Oregon Health and Science University, Portland, OR, United States; 4 Department of Anesthesiology and Critical Care, Perelman School of Medicine, University of Pennsylvania, Philadelphia, PA, United States; 5 Brain Institute, University of California, Los Angeles, Los Angeles, CA, United States

**Keywords:** anatomy, caudoputamen, interneuron, parvalbumin, somatostatin, striatum, tyrosine hydroxylase

## Abstract

**Introduction:**

In addition to spatially organized excitatory forebrain inputs along its mediolateral, dorsoventral, and anteroposterior axes, the dorsal striatum (caudoputamen) relies on cellular diversity to subserve its myriad processing functions. Distinct GABAergic interneuron subtypes, including somatostatin (SST), parvalbumin (PV), and tyrosine hydroxylase (TH) interneurons likely subserve complementary computational roles. However, a detailed understanding of how these microcircuit components are distributed across the caudoputamen remains lacking.

**Methods:**

To address this gap, we generated a comprehensive three-dimensional atlas of genetically-defined SST-, PV-, and TH- labeled interneuron populations across the mouse caudoputamen using genetic labeling, caudoputamen-wide imaging, and voxel-wise quantification.

**Results:**

We found that genetically-defined SST and TH interneurons were relatively enriched in the ventral caudoputamen, whereas PV interneurons were enriched dorsally. In addition, PV and TH interneurons exhibited opposing anteroposterior distribution patterns, with PV interneurons enriched posteriorly and TH interneurons showing a marked decline in density toward the tail of the caudoputamen. Consequently, while the three interneuron subtypes displayed comparable densities in the functionally defined lateral caudoputamen and anterior ventromedial caudoputamen, PV interneurons predominated in the dorsomedial caudoputamen and tail of the caudoputamen. While some statistically significant sex differences were detected, the overall spatial distribution patterns of interneurons were similar across sexes.

**Discussion:**

Together, these findings reinforce the view that the caudoputamen is not a monolithic structure: in addition to excitatory and neuromodulatory inputs, inhibitory microcircuits themselves are differentially distributed across the caudoputamen, providing region-specific constraints on circuit computation. By integrating interneuron organization into existing anatomical frameworks, this atlas provides a foundation for linking dorsal striatal anatomy to function across behavioral domains.

## 1 Introduction

The caudoputamen is the dorsal region of the striatum, the main input nucleus of the basal ganglia. The principal neurons of the caudoputamen—the spiny projection neurons (SPNs)—and sparse cholinergic and heterogeneous GABAergic interneuron subtypes process distributed forebrain projection inputs to support learning, reward processing, movement, and cognition (Choi et al., 2018; Cox and Witten, 2019; Graybiel and Grafton, 2015; Klug et al., 2018; Martel and Galvan, 2022; Wall et al., 2013). Importantly, the caudoputamen is not monolithic. Anatomic tracing studies reveal a striking spatial organization of cortical and thalamic inputs, largely supporting the notion that more dorsolateral regions subserve sensorimotor functions, while more ventromedial regions subserve more limbic functions (Hintiryan et al., 2016; Hunnicutt et al., 2016). In addition to cortical and thalamic inputs, dopaminergic afferents from the ventral midbrain exhibit pronounced topographic organization across the caudoputamen, with regional differences in connectivity, synaptic architecture, and behavioral function (e.g., Lerner et al., 2015; Menegas et al., 2015; Poulin et al., 2018). These findings are bolstered by decades of functional data suggesting that the lateral caudoputamen and medial caudoputamen, respectively, support sensorimotor and associative roles (Alexander et al., 1991; Cox and Witten, 2019). There is also evidence indicating that the anteroposterior axis represents an additional dimension of organization in the caudoputamen, with anterior and posterior territories subserving distinct functions during behavior (Cifuentes et al., 2025; Corbit and Janak, 2010; Mestres-Missé et al., 2012; Yin et al., 2005). Notably, cortical projections do not follow a simple nearest-neighbor mapping along the anteroposterior axis. Instead, distinct projection-defined neuronal subpopulations within a single cortical region preferentially innervate anterior versus posterior territories of the caudoputamen and exhibit dissociable task-related representations (Beckstead, 1979; Choi et al., 2023; Kruttner et al., 2022; Selemon and Goldman-Rakic, 1985), underscoring the AP axis as a meaningful anatomical reference frame with functional relevance (Cifuentes et al., 2025; Shiflett et al., 2010; Wang et al., 2018; Yin et al., 2005).

In addition to spatial organization of inputs, the caudoputamen relies on cellular diversity (Tepper et al., 2018; Tepper et al., 2010) to subserve its myriad processing functions. While the functional properties of SPNs, cholinergic interneurons, and midbrain dopaminergic afferents are comparatively well understood (Apicella, 2017; Cox and Witten, 2019; Lee and Sabatini, 2025), recent work reveals surprising roles for sparse, local circuit GABAergic interneurons in a range of striatal processes. Dendritic-targeting somatostatin (SST) interneurons modulate corticostriatal signaling and learning-related network reorganization (Fino et al., 2018; Holly et al., 2019; Holly et al., 2021; Rotariu et al., 2025; Straub et al., 2016). Soma-targeting parvalbumin (PV) interneurons exert powerful control over spike timing and coordinated ensemble activity (Duhne et al., 2021; Duhne et al., 2024; Duhne et al., 2025; Gritton et al., 2019; Martiros et al., 2018; O’Hare et al., 2017; Owen et al., 2018). A third interneuron class unique to the striatum, Tac2 + nondopaminergic tyrosine hydroxylase (TH) interneurons (Corrigan et al., 2025; Ibanez-Sandoval et al., 2010; Xenias et al., 2015), remains less well characterized, but has been shown to engage local striatal networks and influence behavior (Appings et al., 2024; Ibanez-Sandoval et al., 2010; Kaminer et al., 2019), potentially via inhibitory interactions with SST interneurons (Assous et al., 2017).

While found throughout the caudoputamen, the distribution of these interneuron subtypes is not uniform and not thoroughly described. Previous studies have identified regional biases and planar gradients in interneuron density (Bernácer et al., 2007, 2012; Lecumberri et al., 2017; Del López-González Rey et al., 2022; Luk and Sadikot, 2001; Monteiro et al., 2018; Muñoz-Manchado et al., 2018; Unal et al., 2011; Van Zandt et al., 2024; Wu and Parent, 2000), yet a unified framework of their organization across the full three-dimensional extent of the caudoputamen (as exists for cholinergic interneurons; see Carrasco et al., 2022; Hobel et al., 2026; Matamales et al., 2016) remains lacking. Because distinct interneuron classes regulate different aspects of local circuit function, including dendritic integration and network synchronization, their relative abundance and spatial arrangement could dictate the types of computations made by each territory of the caudoputamen. A detailed understanding of how these microcircuit components are distributed across the caudoputamen therefore provides critical anatomical constraints for interpreting circuit-level function and for contextualizing experimental findings across studies.

To address this gap, we generated a comprehensive three-dimensional atlas of genetically defined SST, PV, and TH interneurons across the mouse caudoputamen (dorsal striatum) using caudoputamen-wide imaging and voxel-wise quantification. This analysis revealed distinct spatial principles governing interneuron organization, including axis-specific gradients and regionally defined subtype compositions. This resource provides an anatomical framework for interpreting and hypothesizing about interneuron function across the caudoputamen.

## 2 Materials and methods

### 2.1 Animals

All procedures and experiments were conducted in accordance with the National Institutes of Health Guidelines for the Use of Animals and approved by the University of Pennsylvania Institutional Animal Care and Use Committee (Protocol: 805643). All mice used in this study were aged 42–43 days at the time of perfusion and were on the C57BL/6 J genetic background.

To label and visualize the spatial distribution of somatostatin (SST), parvalbumin (PV), and tyrosine hydroxylase (TH) interneurons, male mice homozygous for the Cre-reporter line Ai14D (Gt(ROSA)26Sor(tm14(CAG-tdTomato)Hze)/J; Jax 007914) were crossed with female mice expressing either: (1) SST-ires-Cre (Sst(tm2.1(cre)/Zjh)/J; Jax 013044), (2) PV-2a-Cre (Pvalb(tm1.1(cre)Aibs)/J; Jax 012358), or (3) BAC-transgenic TH-Cre (Tg(Th-cre)Fl12Gsat/Mmucd); MMRRC 037415-UCD). This breeding scheme was used to mitigate risk of germline recombination in PV-2a-Cre x Ai14D crosses (Kobayashi and Hensch, 2013). Of note, the TH-Cre line used here is a BAC transgenic line, and animals were genotyped only for presence or absence of the Cre transgene rather than zygosity. Accordingly, we report genotypes as TH-Cre^+^ or TH-Cre^−^ throughout the manuscript.

SST-ires-Cre^+/−^; Ai14D, PV-2a-Cre^+/−^; Ai14D, and TH-Cre^+^; Ai14D offspring were analyzed. Both male and female mice were included. The sensitivity and specificity of SST-ires-Cre and PV-2a-Cre lines for labeling SST + and PV + interneurons in the caudoputamen have been validated previously (Choi et al., 2018). The BAC-transgenic TH-Cre line has been used previously to study striatal TH + interneurons (Kaminer et al., 2019; Xenias et al., 2015).

Throughout, we refer to SST-, PV-, and TH-labeled interneurons as genetically defined populations identified using Cre-driver lines, recognizing that these labels may not perfectly correspond to contemporaneous protein or mRNA expression in all cells.

### 2.2 Tissue preparation for anatomic mapping

Mice were deeply anesthetized via intraperitoneal injection of pentobarbital sodium (150 μL; Sagent, NDC # 25021–676-20). Following loss of the toe-pinch response, mice were transcardially perfused with 15 mL Formalin (10% phosphate-buffered; Fisher Scientific SF100-4) mixed with heparin (50 μL of 1,000 USP units/mL; Meitheal 71,288–402-11). Brains were extracted and post-fixed in Formalin overnight (12–24 h).

Following fixation, brains were sectioned coronally at 50 μm using a Vibratome (5,100 mz; Campden Instruments) in phosphate-buffered saline (PBS). Free-floating sections were serially mounted and coverslipped with Fluoromount-G (Southern Biotech 0100–01) containing DAPI (0.6 μM; Fisher Scientific D1306). Sections were imaged and stitched using a Leica DM6 epifluorescence microscope at 10x magnification. Every single section along the entire anteroposterior extent of the caudoputamen was sectioned, imaged, and used for subsequent analyses.

### 2.3 Immunohistochemistry

Mice were transcardially perfused with 1x phosphate-buffered saline (PBS), followed by 4% paraformaldehyde (PFA). Brains were extracted and post-fixed in 4% PFA for 2 h. Tissue was sectioned coronally at 50 μm using a vibratome (Vibratome, Model 1,000 Plus) in PBS.

Free-floating sections were permeabilized with 0.2% Triton X-100 and blocked for 1 h in 3% normal goat serum (NGS) in PBS. Sections were incubated overnight with primary antibody (rat monoclonal anti-somatostatin, 1:500, Millipore, #MAB354) diluted in PBS containing 1% NGS and 0.2% Triton X-100. After washing, sections were incubated with secondary antibody for 2 h (goat anti-rat IgG (H + L), Alexa Fluor 555 conjugate, 1:500, Invitrogen A21434), then mounted and imaged using an Olympus BX63 epifluorescence microscope at 10x magnification.

### 2.4 3D reconstruction, atlas registration, and cell detection

Following tissue preparation and imaging, exported TIFF files were converted to MBF NeuroInfo-compatible JPEG 2000 format using MBF MicroFile+ and imported into MBF NeuroInfo for all subsequent reconstruction, atlas registration, and cell detection analyses. General data processing workflows using MBF NeuroInfo have been described previously (Eastwood et al., 2018).

Serial sections were assembled and reconstructed using the Serial Section Assembler tool. Individual sections were initially outlined automatically based on image contrast and edge detection, then manually refined as needed. Sections were ordered and aligned to generate a three-dimensional reconstruction, with additional manual refinement performed post-alignment to ensure consistent orientation and section spacing. Three-dimensional reconstructions were saved as JPEG 2000 image stacks (JPX). Every single 50-micron section along the anteroposterior extent of the caudoputamen was used to generate this reconstruction.

Reconstructed brains were manually registered to the Allen Mouse Brain Common Coordinate Framework (CCFv3; Wang et al., 2020) using the Register Sections tool in MBF NeuroInfo (version 2024.1.3). Section angle was determined using the following anatomical landmarks: (1) the section at which the corpus callosum begins to cross the midline, (2) the section in which the rostral anterior commissure appears as three separate components, and (3) the first section in which the fasciculus retroflexus appears as a compact, rounded fiber bundle. The remaining sections were registered automatically and manually refined to maintain consistent alignment and spacing across the entire brain.

Neurons expressing tdTomato were detected using MBF NeuroInfo’s Cell Detection Workflow tool. For SST-Cre; Ai14D brains, image intensity ranges were adjusted (0–500 a.u.) to minimize detection of tdTomato-positive but SST-non-immunoreactive cell clusters, which likely reflect developmentally restricted SST expression (see Supplementary Figure 1). SST-Cre tdTomato-positive cells were detected using a diameter range of 10–15.5 μm and a fixed sensitivity of 11,500 (see Eastwood et al., 2018 for detailed description of this procedure).

**Figure 1:**
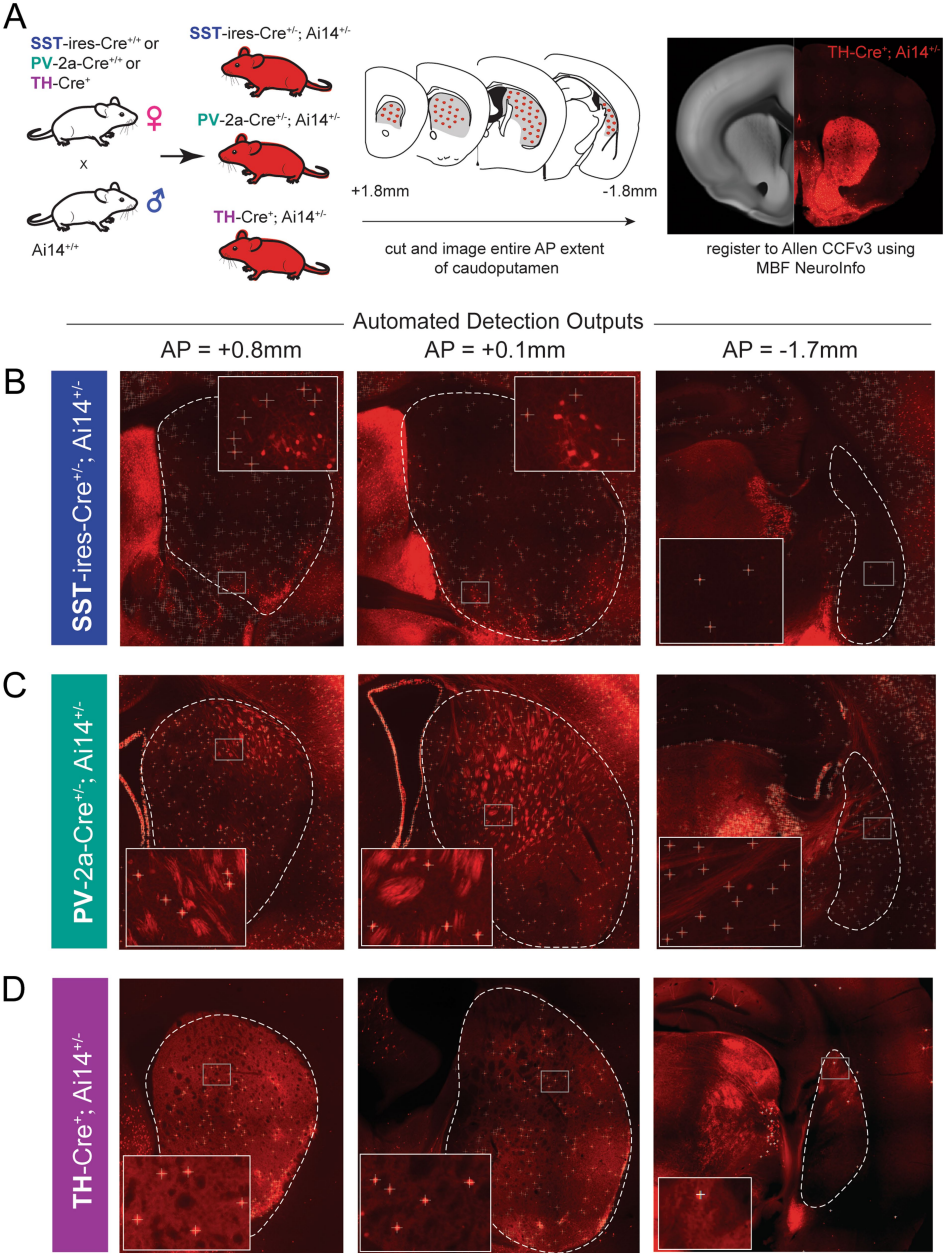
Workflow for genetic identification and imaging of SST, PV, and TH interneurons in the mouse caudoputamen. **(A)** Breeding strategy and overview of tissue preparation, imaging, and atlas registration pipeline. (Left) Mice expressing Cre recombinase in SST, PV, or TH interneurons were crossed to Ai14D reporter mice, and every single coronal section spanning the full anteroposterior extent of the caudoputamen (middle; schematized) was imaged and registered to the Allen Mouse Brain Common Coordinate Framework (CCFv3; Wang et al., 2020) using MBF NeuroInfo (right). **(B–D)** Representative epifluorescence images from three planes of the caudoputamen (AP: +0.8, +0.1, and −1.7 mm relative to bregma) illustrating detection of SST + **(B)**, PV + **(C)**, and TH + **(D)** cells. Caudoputamen extent is delineated by dotted white lines. The lower left inset is a magnification of boxes within the caudoputamen (‘+’ mark detected cells). Detection parameters were optimized separately for each interneuron subtype to minimize false-positive detection. For SST interneurons, settings were adjusted to reduce detection of tdTomato-positive but SST-immunonegative cell clusters (see Supplementary Figure 1). This optimization is illustrated in the insets for the SST sections, illustrating reduced detection of putatively SST-immunonegative cell clusters. For PV interneurons, detection parameters were optimized to minimize inclusion of non-somatic PV + axons and dendrites.

For PV-Cre; Ai14D brains, three separate cell detection workflows were performed per brain to minimize detection of fibers of passage as PV interneurons. These workflows were defined based on reproducible qualitative changes in PV-positive non-somatic process morphology observed across serial sections, including changes in process caliber, density, continuity, and orientation relative to section plane. Specifically, PV-positive non-somatic processes transition from relatively sparse, fine-caliber, and discontinuous profiles to denser, thicker processes that increasingly form continuous fiber-like structures, and ultimately to long, thin processes that run predominantly parallel to the section plane. Boundaries between workflows were determined by visual inspection of these morphological transitions (see Figure 1C). PV-Cre tdTomato-positive cells were detected using a diameter range of 7.5–15 μm, with sensitivities optimized separately for each workflow. For TH-Cre; Ai14D brains, cells were detected using a diameter range of 7–24 μm, with sensitivities optimized on a per-brain basis.

Cell detection outputs were exported for further analysis as CSV files containing per-cell anteroposterior (AP), mediolateral (ML), and dorsoventral (DV) coordinates in Allen CCFv3 reference space (Wang et al., 2020). Individual data files can be shared upon request.

### 2.5 Anatomical data analysis

All subsequent anatomical data analysis was performed in MATLAB using custom scripts, available on the Fuccillo Lab GitHub.^1^ Cell coordinate outputs from MBF NeuroInfo were filtered to include only detections classified as caudoputamen based on Allen CCFv3 taxonomy (Wang et al., 2020), which corresponds to the dorsal striatum (excluding nucleus accumbens and neighboring basal ganglia nuclei such as the globus pallidus). The nucleus accumbens, which constitutes the ventral-most aspect of the striatum, had to be excluded due to the abundance of tdTomato-positive but SST non-immunoreactive clusters of cells in this region. To place all detections within a common anatomical coordinate frame, left hemisphere cell detections were reflected across the midline into the right hemisphere atlas space prior to voxelization and density estimation. This approach allows bilateral pooling while preserving hemisphere identity for lateralization analyses. Hemisphere-specific comparisons did not reveal consistent inter-hemispheric differences (Supplementary Figure 2), supporting the use of mirrored bilateral data for atlas construction.

To estimate cell density, hemisphere-level observations were resampled with replacement (1,000 bootstrapped samples) and binned into cubic voxels (150 × 150 × 150 μm). Voxel-wise densities (cells/mm^3^) were computed separately for each cell type. Voxels were masked using Allen CCFv3 caudoputamen and ventricle masks^2^ and excluded if their Chebyshev distance was less than 50 μm from the caudoputamen boundary or less than 175 μm from the ventricle. The ventricle mask was especially important for exclusion of non-neuronal PV-positive ependymal cells from PV interneuron density estimates. Density maps were smoothed using a Gaussian filter (*σ* = 0.5 voxel).

**Figure 2:**
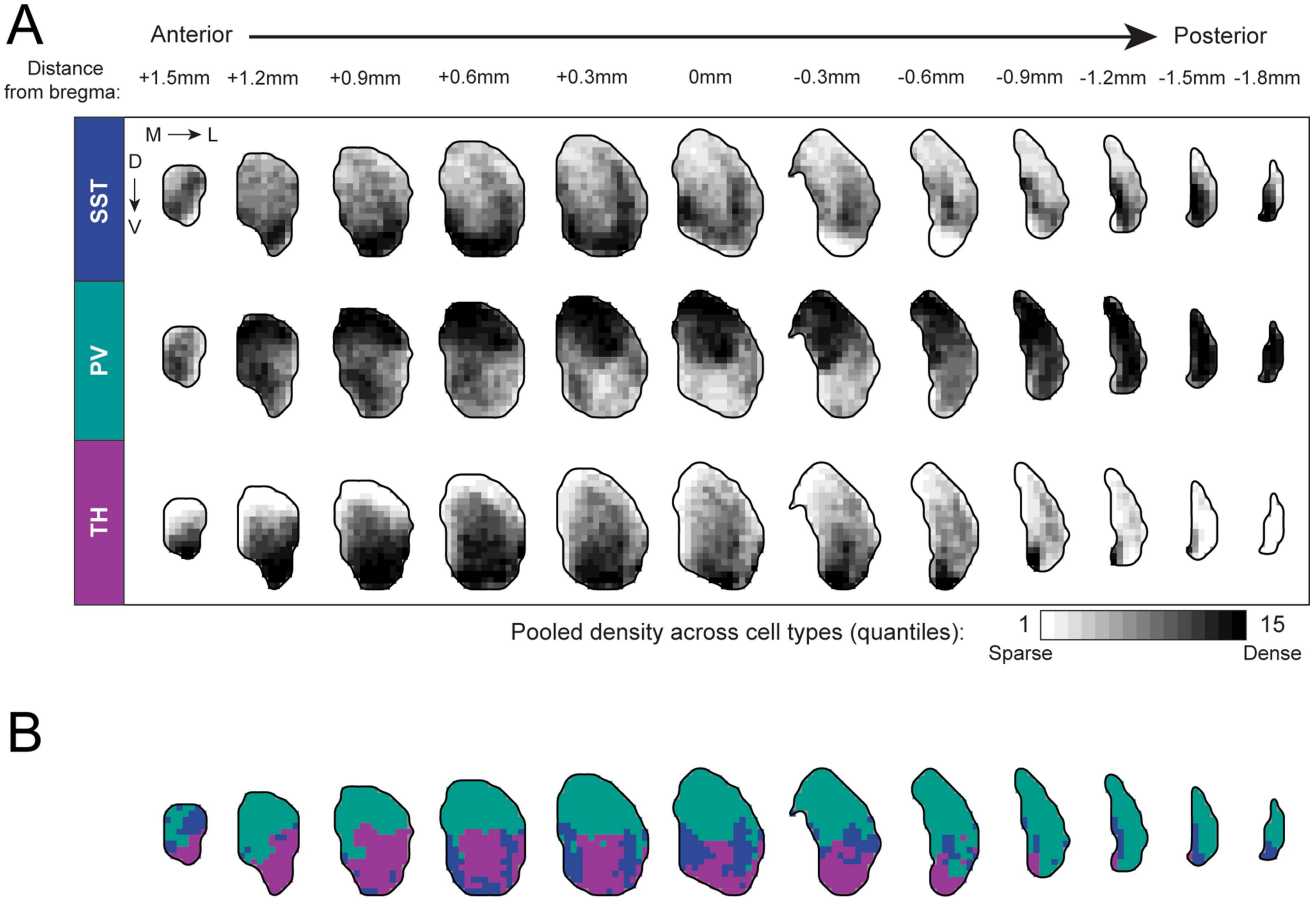
Comprehensive three-dimensional atlas of SST, PV, and TH interneuron distribution across the mouse caudoputamen. **(A)** Voxel-wise density maps showing the spatial distribution of SST (12 hemispheres from 6 animals), PV (11 hemispheres from 6 animals), and TH (13 hemispheres from 7 animals) interneurons across the mouse caudoputamen. The caudoputamen was partitioned into 150 μm voxels, and interneuron densities were computed for each voxel. Density values were subsequently pooled across all cell types to generate 15 quantile-based density thresholds, visualized from sparse to dense. Heatmaps disaggregated by sex are shown in Supplementary Figure 4. **(B)** Voxel-wise predominance maps, in which each voxel was assigned to the interneuron subtype exhibiting the highest density. Voxels are color-coded to indicate the most abundant interneuron subtype (blue, SST; green, PV; purple, TH). This predominance map provides a qualitative summary of relative enrichment and does not imply exclusivity of interneuron subtypes within individual voxels. Predominance maps disaggregated by sex are shown in Supplementary Figure 5.

**Figure 3:**
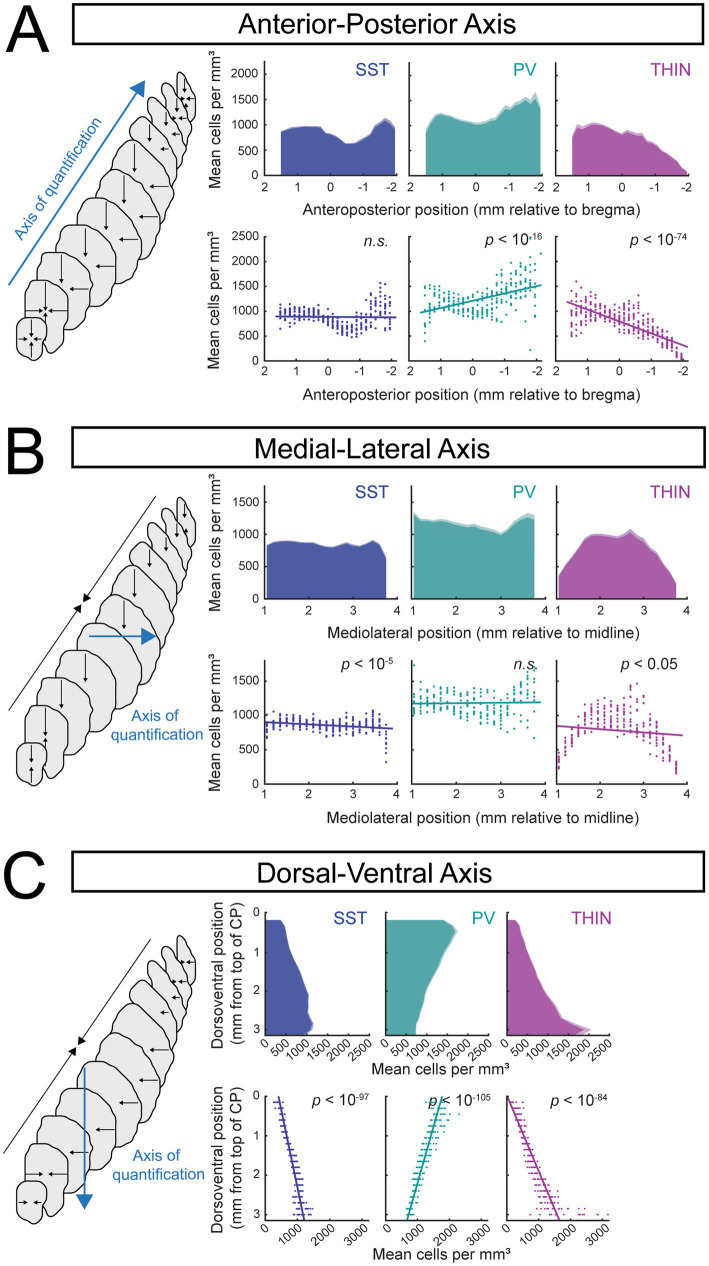
Large-scale spatial gradients of caudoputamen interneuron subtypes across anatomical axes. Voxel-wise interneuron density estimates were quantified along the anterior–posterior **(A)**, medial-lateral **(B)**, and dorsal-ventral **(C)** axes of the mouse caudoputamen. Left schematizes the axis of quantification (blue line) and the axes of compression (black lines). Top rows depict bootstrapped mean density for SST (*N*(hemispheres) = 12), PV (*N*(hemispheres) = 11), and TH (*N*(hemispheres) = 13) across 150-μm planes (1,000 bootstraps per plane, error bars indicate s.e. of the bootstrapped mean). Plots disaggregated by sex are shown in Supplementary Figure 6. Bottom rows show per-hemisphere raw density values for each plane, with overlaid linear mixed-effects model fits. Linear mixed-effects models [Density ~ Coordinate + (1 | Hemisphere_ID)] were used to assess overall directional bias along each anatomical axis. Model slopes represent global directional bias (cells/mm^3^ per mm along each anatomical axis) and are not intended to capture non-monotonic structure. Data disaggregated by sex are shown in Supplementary Figure 7. Anterior–posterior axis. SST: *β* = 4.9 ± 11.1, *p* = 0.66 (*N* = 12 hemispheres); PV: *β* = −146.2 ± 16.1, *p* = 3.0 × 10^−17^ (*N* = 11 hemispheres); TH: *β* = 239.6 ± 9.7, *p* = 3.9 × 10^−75^ (*N* = 13 hemispheres). Medial-lateral axis. SST: *β* = −32.4 ± 7.0, *p* = 6.7 × 10^−6^ (*N* = 12 hemispheres); PV: *β* = 6.5 ± 14.0, *p* = 0.65 (*N* = 11 hemispheres); TH: *β* = −47.5 ± 21.6, *p* = 0.029 (*N* = 13 hemispheres). Dorsal-ventral axis. SST: *β* = 253.9 ± 7.3, *p* = 3.9 × 10^−98^ (*N* = 12 hemispheres); PV: *β* = −350.7 ± 8.7, *p* = 4.0 × 10^−106^ (*N* = 11 hemispheres); TH: *β* = 519.7 ± 17.9, *p* = 5.6 × 10^−85^ (*N* = 13 hemispheres).

Density values were subsequently pooled across all cell types to generate 15 quantile-based density thresholds, which were visualized as heatmaps and voxel-wise predominance heatmaps in Figure 2. Collapsed one-dimensional density profiles were generated for Figure 3, with the standard error of bootstrapped density estimates calculated and overlaid.

**Figure 4:**
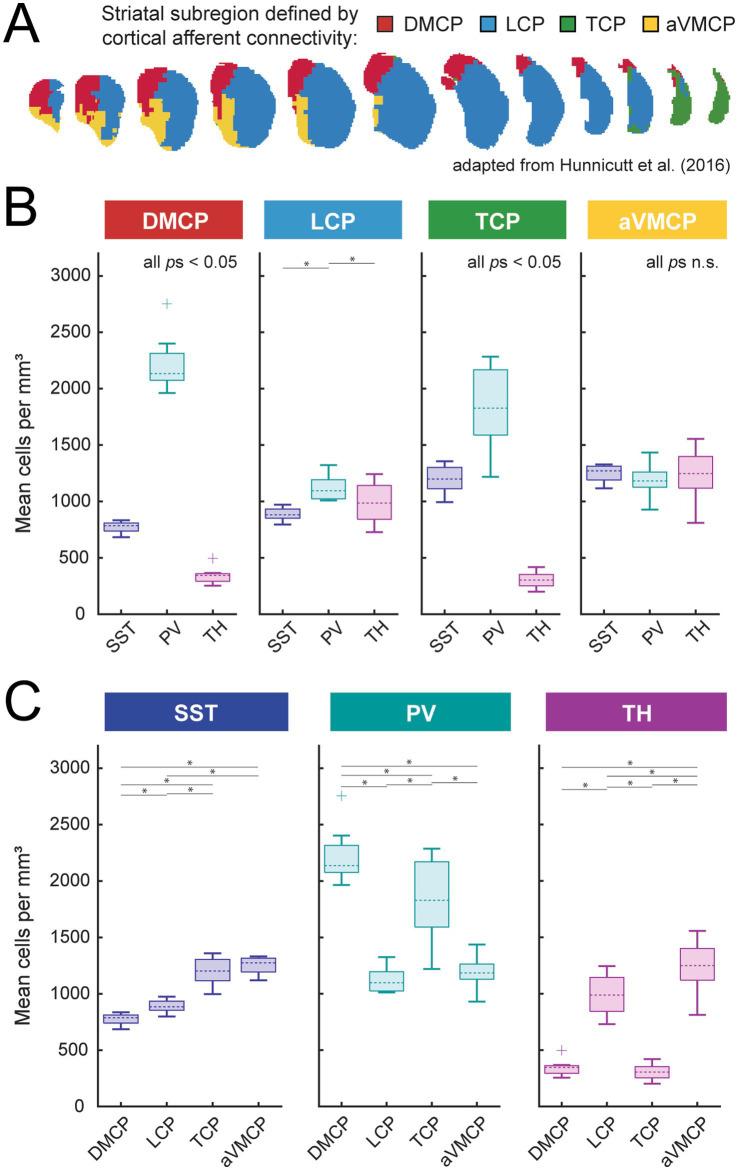
Subregional organization of genetically-identified SST, PV, and TH interneurons of the caudoputamen. **(A)** Schematic adapted from Hunnicutt et al. (2016) illustrating a four-cluster anatomic parcellation of the caudoputamen based on cortical input patterns. Accumbal territories were excluded using a caudoputamen mask. We adopt the following labels: cluster 5 (red), dorsomedial caudoputamen (DMCP); cluster 7 (blue), lateral caudoputamen (LCP); cluster 12 (green), tail of the caudoputamen (TCP); cluster 15 (gold), anterior ventromedial caudoputamen (aVMCP). **(B,C)** Per-hemisphere subregional density distributions (boxplots; center line, median; box, interquartile range; whiskers, non-outlier extrema; points, outliers) are shown organized by subregion **(B)** or by interneuron subtype **(C)** (SST – 12 hemispheres from 6 animals, PV – 11 hemispheres from 6 animals, TH – 13 hemispheres from 7 animals). Mixed-effects ANOVAs [linear mixed-effects models with random intercept for hemisphere; (1 | Hemisphere_ID)] were used to test (i) subtype differences within each subregion [Density ~ Subtype + (1 | Hemisphere_ID)] and (ii) subregional differences within each subtype [Density ~ Subregion + (1 | Hemisphere_ID)]. Sidak-corrected significant pairwise comparisons (*p* < 0.05) are marked with asterisks. Omnibus subtype effects within subregion (panel B): DMCP: *p* = 2.1 × 10^−22^; LCP: *p* = 3.9 × 10^−4^; TCP: *p* = 2.7 × 10^−14^; aVMCP: *p* = 0.32 (n.s.). Omnibus subtype effects within subtype (panel C): SST: *p* = 2.1 × 10^−15^; PV: *p* = 6.5 × 10^−13^; TH: *p* = 6.4 × 10^−22^.

Cell coordinates were additionally assigned to one of four subregions of the caudoputamen (see Figure 4A) based on previously defined clusters from Hunnicutt et al. (2016), available in Allen CCFv3 space on GitHub.^3^ These subregions were transformed into Allen CCFv3 space and masked using the Allen CCFv3 caudoputamen mask to generate subregion volumes. Per-hemisphere densities (cells/mm^3^) were calculated for each cell type and subregion and visualized as boxplots (Figure 4B).

### 2.6 Statistical methods

To quantify the large-scale spatial organization of interneuron density along major dorsal striatal axes, voxel-wise densities were projected onto the corresponding anatomical coordinates and analyzed using linear mixed-effects models. This approach was chosen to provide a parsimonious, directionally interpretable summary of axis-level bias, rather than to model fine-scale spatial structure.

First-pass models included anatomical coordinate as the sole fixed effects predictor, with hemisphere identity included as a random intercept to account for repeated voxel measurements across hemispheres:


$$\text{Voxelwise density}=\beta_{0}+\beta_{1}\text{Coordinate}+u_{\text{Hemisphere}}+\varepsilon$$

Follow-up models to investigate sex differences included anatomical coordinate, sex, and their interaction as fixed effects, with hemisphere identity included as a random intercept to account for repeated voxel measurements across hemispheres:


$$\begin{array}{l} \text{Voxelwise density}=\beta_{0}+\beta_{1}\text{Coordinate}+\beta_{2}\mathrm{Sex}\\ +\beta_{3}\;(\text{Coordinate}\times \mathrm{Sex})+u_{\text{Hemisphere}}+\varepsilon \end{array}$$

Models were fit using the *fitlme* function in MATLAB [formula first-pass: “Density ~ Coordinate * Sex + (1 | Hemisphere_ID)”; formula follow-up: “Density ~ Coordinate * Sex + (1 | Hemisphere_ID)”].

A similar approach was used to examine differences in interneuron composition across cortical projection-defined territories of the caudoputamen, both within subregions (cell-type composition) and across subregions (regional enrichment). In these analyses, interneuron density was modeled as a function of either interneuron subtype (for within-subregion comparisons), or subregion (for within-subtype comparisons), together with sex and their interactions in follow-up models, with hemisphere identity included as a random intercept.

## 3 Results

To characterize the distribution of genetically defined somatostatin (SST), parvalbumin (PV), and tyrosine hydroxylase (TH) expressing interneruons throughout the mouse caudoputamen, we generated a three-dimensional atlas using genetic labeling, caudoputamen-wide imaging, and voxel-wise quantification. To this end, mice homozygous for SST-Cre, PV-Cre, or TH-Cre were crossed to the mice homozygous for the Cre-dependent reporter line Ai14D (Figure 1A), yielding offspring in which targeted interneuron populations expressed the red fluorophore tdTomato.

At post-natal day 42–43, mice were sacrificed, and all coronal 50-micron sections containing the caudoputamen were imaged and registered to the Allen Common Coordinate Framework v3 (CCFv3; Wang et al., 2020) using MBF NeuroInfo (full detains in Methods). The built-in Cell Detection Workflow was used to identify tdTomato-positive somata and map their spatial coordinates into CCFv3 space (Figures 1B–D).

Although the SST-ires-Cre line has previously been used to target SST interneurons in the caudoputamen (Choi et al., 2018; Holly et al., 2019), we observed discrete clusters of dense tdTomato-positive somata largely in ventrolateral caudoputamen that lacked immunoreactivity for SST (Supplementary Figure 1). These observations indicate that SST-Cre labeling may capture cells with developmental or otherwise noncanonical SST-lineage labeling. Regardless, to ensure conservative quantification, detection parameters were optimized to minimize the contribution of these clusters to our final cell counts (see Figure 1B for an example of excluded signal, and Supplementary Figure 1 for a detailed description of the exclusion procedure). The results of this more conservative detection approach broadly corresponded to the anatomical pattern of *Sst in situ* hybridization data from the Allen Mouse Brain Atlas (Supplementary Figure 3) (Allen Institute for Brain Science, 2004).^4^

In our PV-2a-Cre; Ai14D crosses, we additionally observed tdTomato-positive fiber-like structures lacking identifiable somata, with morphology and density that varied across the anteroposterior axis of the caudoputamen. These structures were distinct from the compact, round profiles characteristic of neuronal cell bodies and likely reflect labeling of axonal processes from PV-expressing populations outside the caudoputamen. To prevent contamination of voxel-wise cell density estimates, detection parameters were optimized to selectively exclude non-somatic signal while preserving bona-fide tdTomato-positive cell bodies (see Figure 1C; Supplementary Figure 1).

In our TH-Cre; Ai14D crosses, we observed background fluorescence in the caudoputamen arising from dopaminergic afferents originating in ventral midbrain nuclei. To improve visualization of sparsely distributed tdTomato-positive interneuron somata, image display histograms were adjusted using standard linear contrast scaling to enhance cellular contrast.

### 3.1 Voxel-wise three-dimensional map of GABAergic interneuron distributions

Cells were detected and mapped to Allen Common Coordinate Framework (CCFv3; Wang et al., 2020) using MBF NeuroInfo. To generate a three-dimensional map of genetically-defined SST, PV, and TH interneuron distributions in the caudoputamen, we partitioned the Allen CCFv3 caudoputamen into 150-μm isotropic voxels (3,177 150-μm voxels per caudoputamen, corresponding to a volume of roughly 10.7 mm^3^) and computed the voxel-wise interneuron densities across the caudoputamen [Figure 2; SST (12 hemispheres from 6 animals, PV (11 hemispheres from 6 animals), and TH (13 hemispheres from 7 animals)].

SST interneurons exhibited a non-uniform distribution, with relative enrichment in the ventral portion of caudoputamen, and particularly within the anterior dorsomedial caudoputamen (DMCP), as well as additional regions of elevated density along the lateral caudoputamen. SST interneurons were also prominent within the medial tail of the caudoputamen (Figure 2B, top). In contrast, PV interneurons were distributed broadly throughout the dorsal caudoputamen across anteroposterior levels, with comparatively lower density in ventral regions of the caudoputamen. Notably, PV density remained high in posterior caudoputamen and extended throughout the tail of the caudoputamen (Figure 2B, middle). TH interneurons showed the most circumscribed spatial profile, with very sparse representation in dorsal- and medial-most aspects of the caudoputamen, and near absence from the tail of the caudoputamen. Instead, the highest TH interneuron densities were observed in ventral and more anterior planes of the caudoputamen (Figure 2B, bottom).

In anterior sections, SST and TH interneurons displayed partially overlapping ventral enrichment patterns that contrasted with more dorsally biased distribution of PV interneurons. This organization progressively diminished in posterior planes as TH interneuron density declined (Figure 2B).

These spatial relationships are summarized using a voxel-wise predominance map (Figure 2C) in which each voxel is assigned to the interneuron subtype exhibiting the highest density. This representation highlights the predominance of PV interneurons in the dorsal caudoputamen, relative enrichment of SST interneurons in ventral and posterior regions of the caudoputamen, and preferential representation of TH interneurons in anterior and ventral caudoputamen. Importantly, the predominance map is intended as a qualitative summary of relative enrichment and does not imply exclusivity of interneuron subtypes within individual voxels. Atlas and predominance maps disaggregated by sex are provided in Supplementary Figures 4, 5.

### 3.2 Interneuron organization along major anatomic axes

To quantify large-scale spatial organization of genetically-defined interneuron subtypes, we examined how SST, PV, and TH interneuron densities vary along the anteroposterior (AP), mediolateral (ML), and dorsoventral (DV) axes of the caudoputamen (Figure 3). To address whether interneurons were preferentially enriched toward one extreme of an anatomical axis relative to the other, voxel-wise densities were projected onto the corresponding anatomical coordinates (blue ‘axis of quantification’) and analyzed using linear mixed-effects models. This approach was chosen to provide a parsimonious, directionally interpretable summary of axis-level bias, rather than to model fine-scale spatial structure. Because several distributions exhibited clear non-monotonic features, model slopes were interpreted as measures of global directional tendency rather than complete descriptions of spatial patterning.

#### 3.2.1 Anteroposterior axis

Along the anteroposterior axis, both SST and PV interneurons exhibited non-uniform distributions characterized by a local reduction in density at intermediate levels of the caudoputamen, and a marked increase toward posterior, tail-associated regions (Figure 3A). This posterior enrichment was particularly prominent for PV interneurons. Interestingly, the increase in SST cell density toward the tail was restricted to more medial parts of this structure. Consistent with this pattern, linear mixed-effects modeling revealed a significant overall increase in PV density along the anteroposterior axis (*β* = −146.2, *p* = 3.0 × 10^−17^, *N*(hemispheres) = 11, *N*(sections) = 297), whereas SST interneurons did not exhibit a significant monotonic change across the axis (*β* = 4.9, *p* = 0.66, *N*(hemispheres) = 12, *N*(sections) = 324).

In contrast, TH interneurons displayed a pronounced decrease in density toward posterior levels of the caudoputamen (Figure 3A; *β* = 239.6, *p* = 3.9 × 10^−75^, *N*(hemispheres) = 13, *N*(sections) = 351), consistent with atlas-level distributions shown in Figure 2B. This posterior decline was significantly steeper in males than females (*β*_female = 212.9, *β*_male = 270.75, sex x position *β* = 57.85; *p* = 0.00278). Sex-disaggregated data are provided in Supplementary Figures 6, 7.

#### 3.2.2 Mediolateral axis

Along the mediolateral axis, SST interneurons exhibited relatively uniform density, with a small but statistically significant negative slope (Figure 3B; *β* = −32.4, *p* = 6.7×10^−6^, *N*(hemispheres) = 12, *N*(sections) = 264), indicating subtle lateral depletion rather than a strong spatial gradient.

PV interneurons showed a clearly non-monotonic mediolateral distribution (Figure 3B). In line with this nonlinear structure, linear mixed-effects modeling did not detect a significant overall slope across the mediolateral axis (*β* = 6.5, *p* = 0.65, *N*(hemispheres) = 11, *N*(sections) = 242). Notably, males and females exhibited divergent trends across the mediolateral axis (*β*_female = −61.34, *β*_male = 62.96, sex x position interaction *β* = 124.3, *p* = 5.66 × 10^−6^), with sex-disaggregated data distributions shown in Supplementary Figures 6, 7.

TH interneurons displayed reduced density at both the most medial- and lateral-most planes, with higher density in intermediate mediolateral positions (Figure 3B), consistent with atlas-level observations (Figure 2B). This nonlinear pattern yielded a small but statistically significant negative slope in linear mixed-effects modeling (*β* = −47.5, *p* = 0.029, *N*(hemispheres) = 11, *N*(sections) = 242), likely reflecting reduced TH density in tail regions.

#### 3.2.3 Dorsoventral axis

Along the dorsoventral axis, both SST and TH interneurons exhibited robust ventral enrichment. TH interneuron density increased steeply toward ventral caudoputamen (*β* = 519.7, *p* = 5.6 × 10^−85^, *N*(hemispheres) = 13, *N*(sections) = 299), while SST interneurons showed a more moderate but highly significant ventral increase (*β* = 253.9, *p* = 3.9 × 10^−98^, *N*(hemispheres) = 12, *N*(sections) = 276). The dorsoventral TH gradient was significantly more pronounced in males than females (*β*_female = 441.6, *β*_male = 610.8, sex x position interaction *β* = 169.2, *p* = 1.54 × 10^−6^); sex-disaggregated data are shown in Supplementary Figures 6, 7.

In contrast, PV interneurons exhibited a strong decrease in density along the dorsoventral axis (*β* = −350.7, *p* = 4.0 × 10^−106^, *N*(hemispheres) = 11, *N*(sections) = 253), indicating preferential dorsal localization relative to SST and TH interneurons.

### 3.3 Subregion-level organization of interneuron subtypes

Another approach to quantify large-scale spatial gradients was with regard to cortical afferent-defined territories (as previously examined in Hunnicutt et al., 2016). Voxels in the caudoputamen were assigned to sub-clusters ‘dorsomedial caudoputamen’ (DMCP), ‘lateral caudoputamen’ (LCP), ‘tail of the caudoputamen’ (TCP), and ‘anterior ventromedial caudoputamen’ (aVMCP) clusters based on dominant cortical afferent identity derived from Allen Institute connectivity mapping studies (Figure 4A). Linear mixed-effects models were used to assess differences in interneuron density both within defined subregions (cell-type composition; Figure 4B) and across subregions (regional enrichment; Figure 4C).

#### 3.3.1 Within-subregion interneuron composition

Within subregions of the caudoputamen, interneuron composition was broadly similar. In both the dorsomedial caudoputamen (DMCP) and the tail of the caudoputamen (TCP), PV interneurons were the most abundant subtype, SST interneurons were present at intermediate densities, and TH interneurons were sparsest (Figure 4B), consistent with the dorsal bias observed in axis-based analyses.

In the lateral caudoputamen (LCP), subtype distinctions were less pronounced. PV enrichment was reduced, and SST and TH interneurons were present at more comparable densities. A significant cell type x sex interaction [*F* (2,30) = 4.8226, *p* = 0.015], was driven by a greater density of TH interneurons in males [mean density = 1087.6 cells/mm^3^; 95% CI (887.6, 1287.5)] than in females [mean density = 904.4 cells/mm^3^; 95% CI (803.4, 1005.4)], as shown in Supplementary Figures 8, 9. In males, TH and PV interneurons were similarly abundant (*p*_Sidak_ = 1), and each exceeded SST interneuron density (*p*s_Sidak_ < 0.03), whereas females followed the general population pattern with PV predominance.

In the anterior ventromedial caudoputamen (aVMCP), interneuron densities were comparatively balanced across SST, PV, and TH subtypes. A significant cell type x sex interaction [*F* (2,30) = 4.9432, *p* = 0.014] reflected greater TH interneuron density in males [mean density = 1364.2 cells/mm^3^; 95% CI (1239.3, 1489.2)] than in females [m(density) = 1163.1 cells/mm^3^; 95% CI (935.4, 1390.7)], as shown in Supplementary Figures 8, 9. In males, TH interneurons were significantly more abundant than PV interneurons (*p*_Sidak_ < 0.005), whereas no subtype-specific differences were detected in females.

#### 3.3.2 Across-subregion comparisons

Across territories of the caudoputamen, interneuron subtypes exhibited distinct regional biases (Figure 4C). SST interneurons were most abundant in aVMCP and TCP and were sparsest in DMCP. PV interneurons reached their highest densities in DMCP and TCP and were less abundant in LCP and aVMCP. In contrast, TH interneurons displayed a complementary distribution relative to PV interneurons, with lower density in DMCP and TCP and greater representation in LCP and aVMCP.

Together, these results indicate that continuous spatial gradients in interneuron density resolve into distinct subtype compositions across cortical afferent-defined striatal territories. Thus, large-scale anatomical organization of interneuron populations is reflected not only along continuous coordinate axes, but also in discrete functional architecture defined by cortical input domains.

## 4 Discussion

In this study, we generated a three-dimensional atlas of genetically defined somatostatin- (SST), parvalbumin- (PV), and tyrosine hydroxylase-labeled (TH) interneuron populations in the caudoputamen (dorsal striatum) of mice using genetic labeling, caudoputamen-wide imaging, and voxel-wise analyses. We quantified gradients of distribution using linear-mixed effects models and compared interneuron densities across cortical afferent-defined subregions of the caudoputamen, finding striking differences in the distribution of the three interneuron subtypes. Most notably, we found that SST and TH interneurons were relatively enriched ventrally, whereas PV interneurons were enriched dorsally. PV and TH interneurons also had opposite anteroposterior distribution patterns, with PV interneurons enriched posteriorly while TH interneurons showed a significant decline in density posterior to bregma and were nearly absent from the tail of the caudoputamen. Thus, whereas the three interneuron subtypes had comparable densities in the lateral caudoputamen (LCP) and anterior ventromedial caudoputamen (aVMCP), PV interneurons predominated in the dorsomedial caudoputamen (DMCP) and tail of the caudoputamen (TCP). While some statistically significant sex differences were detected, the overall spatial distribution patterns of interneurons were similar across sexes.

In several respects, our findings align with previously published literature. We recapitulate a dorsal-to-ventral gradient of decreasing PV interneuron density (Del López-González Rey et al., 2022; Luk and Sadikot, 2001; Tepper et al., 2010), as well as an anteroposterior gradient of increasing PV interneuron density, consistent with observations in macaques (Del López-González Rey et al., 2022) but differing from earlier findings in rats (Wu and Parent, 2000). Importantly, the latter study did not include the tail of the caudoputamen; in our dataset, a pronounced increase in PV interneuron density in this region was the primary contributor to the observed anteroposterior gradient, likely accounting for the discrepancy between studies. We additionally observed a dorsal-to-ventral gradient of increasing density in TH interneurons, as previously described in macaques (Del López-González Rey et al., 2022). To our knowledge, no studies have quantitatively assessed distribution gradients of SST interneurons across the caudoputamen. However, one recent study found that SST-immunoreactive cells are more abundant in the nucleus accumbens than in the caudoputamen (Van Zandt et al., 2024), hinting at a dorsal-to-ventral gradient of increasing density as observed in our analysis. Although SST-Cre labeling in our dataset was confounded by SST-immuno-negative tdTomato+ clusters in the ventral striatum, particularly in the nucleus accumbens thus precluding its inclusion, ventral enrichment of genetically defined SST interneurons was preserved within the caudoputamen after exclusion of these artifacts.

Some aspects of our findings diverge from prior anatomic studies, however. With our recombinase-based genetic strategy, we do not recapitulate the medial-to-lateral gradient of increasing density of PV interneurons that has been reported in immunohistochemical (IHC) studies in mice (Monteiro et al., 2018), rats (not Garas et al., 2016; but Gerfen, 1985; Wu and Parent, 2000), squirrel monkeys (Wu and Parent, 2000), macaques (Del López-González Rey et al., 2022), and humans (Bernácer et al., 2012). Because our approach provides a binarized readout of PV expression, rather than quantitative information about expression levels, one possible explanation for this discrepancy is that IHC studies may be sensitive to gradients in PV expression rather than differences in absolute interneuron number (Monteiro et al., 2018). This interpretation is supported by single-cell RNA-sequencing analyses, demonstrating that *Pvalb* is highly co-expressed with *Pthlh*, and that neurons expressing these markers constitute a shared PV/PTHLH transcriptional class (Muñoz-Manchado et al., 2018). Within this class, *Pthlh* transcript levels decrease along the mediolateral axis, contrasting with an increase in *Pvalb* transcript density along the same dimension. Moreover, genetic labeling in PV-Cre mice captured interneurons that were *Pthlh+* but *Pvalb-* by *in situ* hybridization, suggesting that IHC or ISH for *Pvalb* alone might fail to identify even genetically labeled interneurons in this class. One explanation for the identified PV gradient is that *Pvalb* expression levels scale with electrophysiological demands associated with rapid excitatory drive. Such an interpretation is borne out by Patch-Seq in this same study (Muñoz-Manchado et al., 2018), which finds that higher *Pvalb* transcript levels were correlated with lower action potential half-width and higher maximal firing rates in the PV/PTHLH class. Together, these findings raise the possibility that mediolateral gradients observed in PV IHC studies reflect variation in PV expression levels within a distributed interneuron class, rather than differences in absolute cell density.

Because distinct interneuron classes regulate different aspects of local circuit function, including dendritic integration and network synchronization, their relative abundance and spatial arrangement could dictate the types of computations that predominate in each region of the caudoputamen. Accordingly, a detailed understanding of how these microcircuit components are distributed across the caudoputamen therefore provides important anatomical constraints for interpreting circuit-level function and for contextualizing experimental findings across studies. For example, dendritic-targeting somatostatin (SST) interneurons modulate corticostriatal signaling and learning-related network reorganization (Fino et al., 2018; Holly et al., 2019; Holly et al., 2021; Rotariu et al., 2025; Straub et al., 2016), while soma-targeting parvalbumin (PV) interneurons exert powerful control over spike timing and coordinated ensemble activity (Duhne et al., 2021; Duhne et al., 2025; Gritton et al., 2019; Martiros et al., 2018; O’Hare et al., 2017; Owen et al., 2018). The relative enrichment of PV interneurons in the dorsal caudoputamen may therefore reflect greater computational demands for precise regulation of ensemble dynamics in sensorimotor processing. Conversely, the increased representation of SST interneurons in ventral regions of the caudoputamen may be consistent with heightened demands on regulation of dendritic excitability and synaptic plasticity in territories canonically associated with reward-processing and learning. However, it is important to note that these constraints are not deterministic. While interneuron distributions may inform hypotheses about the relative computational capacities of different subregions of the caudoputamen, they are not prescriptive. For example, even in regions where particular interneuron subtypes are comparatively sparse, their density remains non-null, indicating that regional differences are quantitative rather than categorical. Furthermore, specific interneuron subtypes may exhibit synaptic connectivity at a distance from their cell bodies (Straub et al., 2016). Ultimately, *in vivo* recordings and manipulations will be necessary to discern functional contributions of interneuron subtypes (e.g., Duhne et al., 2025; Holly et al., 2019; Lee et al., 2017; Owen et al., 2018).

Our study has several limitations that should be considered. Firstly, the use of recombinase-based genetic strategies carries the inherent risk of labeling neurons with developmentally restricted gene expression that are not bona fide members of the interneuron class of interest. For example, in our SST-Cre; Ai14D mice, we observed dense clusters of tdTomato+ cells that were not SST-immunoreactive and whose distribution and morphology were inconsistent with prior descriptions of striatal SST interneurons. These clusters were particularly abundant in the nucleus accumbens, the ventral-most territory of the striatum, which precluded its inclusion in our anatomic atlas. Accordingly, our atlas should be interpreted primarily as a map of genetically labeled interneuron populations, rather than a definitive immunohistochemical census of all SST-, PV-, and TH-expressing cells. Future work should use alternative approaches to characterize interneuron distributions in this ventral-most region of the anterior striatum. In addition, genetic labeling results in expression within extrinsic axonal or dendritic processes, as well as occasional non-neuronal cell types, which can complicate automated cell detection and quantification. This issue was most pronounced in our PV-Cre; Ai14D mice, where periventricular ependymal cells and dense PV + axons necessitated masking and more rigorous detection methods to isolate PV + somata. Although we implemented conservative exclusion criteria, it remains possible that these factors introduced bias into density estimates.

While our TH-Cre; Ai14D line did not exhibit such issues, the high density of TH + dopamine neuron axons made it impossible for us to immunohistochemically validate the sensitivity and specificity of this line to TH interneurons. However, the TH-Cre line used here has been widely employed in prior studies of striatal TH interneurons (Assous et al., 2017; Kaminer et al., 2019; Xenias et al., 2015), and the distribution patterns we observed—including ventral enrichment and relative sparing of the tail and dorsal- and medial-most aspects of the caudoputamen—closely mirror *Th* mRNA expression patterns in the Allen Mouse Brain Atlas (Lein et al., 2006).

Additional methodologic limitations include reconstruction of three-dimensional distribution patterns from sectioned tissue, lack of intersectional genetic or immunohistochemical validation, and reliance on SST, PV, and TH as primary markers of interneuron identity. Future work could leverage tissue clearing and light sheet imaging approaches (e.g., CLARITY, iDISCO), combined with whole-brain immunohistochemistry or *in situ* hybridization for complementary markers, including nNOS, NPY, and CHODL for SST interneurons; PTHLH for PV interneurons, TAC2 for TH interneurons, and GAD67 for all, to provide more robust classification of interneuron subtypes. In addition, our analysis did not address the compartmental organization of interneurons within striosomal and matrix domains, another important principle of striatal organization (Brimblecombe and Cragg, 2017; Graybiel and Matsushima, 2023). For example, TH interneurons have been reported to preferentially localize to striosomes in ventral striatum (Unal et al., 2011), a feature we were unable to evaluate here. Future studies integrating interneuron mapping with markers such as the *μ*-opioid receptor may help resolve how interneuron distributions interact with striatal compartmentalization. Finally, our distribution map across four projection-identified subclusters (Hunnicutt et al., 2016) represents only one possible taxonomy of the caudoputamen. Alternative schemes have been proposed (Hintiryan et al., 2016), including a recent subdivision of the rodent caudoputamen into tail of caudate (CaT) and caudal putamen (PuC) based on gene expression patterns and afferent connectivity (Ma et al., 2025). Incorporating such frameworks will be an important future direction.

Together, these findings reinforce the view that the caudoputamen is not a monolithic structure but is instead organized along multiple spatial dimensions that shape how information is locally processed. In addition to structured excitatory inputs, inhibitory microcircuits themselves are differentially distributed across the caudoputamen, providing region-specific constraints on circuit computation. By integrating these microcircuit features into existing anatomical frameworks, this atlas provides a foundation for linking anatomy to function across behavioral domains.

## Data Availability

The datasets presented in this study can be found in online repositories. The names of the repository/repositories and accession number(s) can be found at: https://zenodo.org/records/18685812.

## Footnotes

1
https://github.com/Fuccillo-Lab

2 structures 672 and 73 respectively, available at https://download.alleninstitute.org/informatics-archive/current-release/mouse_ccf/annotation/ccf_2017/structure_masks/structure_masks_50/

3
https://github.com/TianyiMaoLab/striatumclusters

4
mouse.brain-map.org/experiment/show/1001
